# Messenger Ribonucleic Acid (mRNA)-Based Universal Vaccines: Engineering Broad-Spectrum Immunity Against Future Pandemics

**DOI:** 10.7759/cureus.84821

**Published:** 2025-05-26

**Authors:** Joan C Muodiaju, Chidinma S Madu

**Affiliations:** 1 Surgery, Duke University School of Medicine, Durham, USA; 2 Health Sciences, Duke University Health System, Durham, USA

**Keywords:** covid-19, immune response, infectious diseases, mrna vaccine, pandemic preparedness, vaccine development

## Abstract

The rapid emergence and evolution of infectious pathogens, including the COVID-19 pandemic and recurring influenza outbreaks, underscore the need for universal vaccines capable of providing broad-spectrum immunity. Messenger ribonucleic acid (mRNA) vaccine technology has emerged as a transformative platform due to its rapid development, high immunogenicity, and adaptability to new variants. Unlike conventional vaccines, which rely on weakened or inactivated pathogens, mRNA vaccines instruct host cells to produce antigens that elicit robust immune responses. This paper explores the design principles, mechanisms of action, and advancements in mRNA-based universal vaccines, emphasizing their potential against influenza, coronaviruses, and antimicrobial-resistant pathogens. We discuss innovations such as self-amplifying mRNA (saRNA), nanoparticle-based delivery systems, and artificial intelligence (AI)-driven antigen selection. Additionally, challenges such as antigenic variability, immune evasion, stability issues, and global distribution barriers are addressed. With continued research and development, mRNA-based universal vaccines could play a critical role in pandemic preparedness and global health security.

## Introduction and background

Infectious diseases have posed significant public health challenges throughout history, with periodic outbreaks of influenza, coronaviruses, and antimicrobial-resistant pathogens. The COVID-19 pandemic, caused by SARS-CoV-2, demonstrated the limitations of conventional vaccine platforms, which require lengthy development timelines and are often specific to individual strains [1]. Traditional vaccines, such as live-attenuated and inactivated vaccines, provide effective immunity but require continuous updates due to antigenic drift and shift, particularly in influenza and coronavirus strains [2,3].

mRNA vaccines offer a groundbreaking alternative by utilizing synthetic genetic material to encode antigens directly within host cells. The rapid development and high efficacy of the Pfizer-BioNTech (New York, USA) and Moderna COVID-19 (Massachusetts, USA) vaccines have highlighted the potential of mRNA technology in pandemic response [3]. This paper explores the feasibility of mRNA-based universal vaccines, which aim to provide long-lasting, broad-spectrum immunity against evolving infectious diseases.

Objectives and scope

The primary objective of this paper is to explore the transformative potential of mRNA-based technologies in the development of universal vaccines capable of providing broad-spectrum immunity against a wide range of infectious diseases. Specifically, the paper reviews the scientific principles, mechanisms, design strategies, and technological innovations that support the feasibility of mRNA platforms in addressing global health threats, including rapidly mutating viruses and antimicrobial-resistant pathogens.

This article synthesizes emerging evidence and highlights the comparative advantages of mRNA vaccines over traditional platforms, highlighting their speed of development, adaptability to emerging variants, and ability to stimulate strong and diverse immune responses. In addition, it investigates novel innovations such as self-amplifying mRNA (saRNA), nanoparticle-based delivery systems, and the integration of artificial intelligence (AI) and machine learning in antigen selection and vaccine optimization.

The scope of this paper encompasses a comprehensive review of the immunological mechanisms of mRNA vaccines, the design and development strategies behind universal vaccine candidates, and their applications in combating high-priority pathogens such as influenza, SARS-CoV-2, HIV, and antimicrobial-resistant bacteria. Furthermore, the paper discusses ongoing challenges, such as cold chain requirements, durability of immunity, and immune evasion, and provides forward-looking perspectives on future research directions aimed at improving mRNA vaccine efficacy, stability, and global accessibility. Through this multidisciplinary lens, the paper aims to contribute to the growing body of literature supporting mRNA-based universal vaccines as a cornerstone of future pandemic preparedness and global immunization strategies.

The science of mRNA vaccine technology

Messenger RNA (mRNA) vaccines represent a significant advancement in immunization strategies, offering a novel approach distinct from traditional vaccine platforms. Unlike conventional vaccines that often utilize inactivated or attenuated pathogens to elicit an immune response [4], mRNA vaccines employ synthetic mRNA sequences to instruct host cells to produce specific antigenic proteins. These proteins subsequently stimulate both humoral (B-cell) and cellular (T-cell) immune responses, providing robust protection against targeted pathogens [2-5].

The synthetic mRNA used in vaccines incorporates optimized untranslated regions (UTRs), codon optimization, and a 5′ cap and poly(A) tail to enhance stability and translational efficiency [5]. Additionally, the mRNA sequence is often chemically modified to evade innate immune detection, thus enhancing antigen expression and minimizing inflammatory responses [6].​

Mechanism of mRNA vaccines

**Figure 1:**
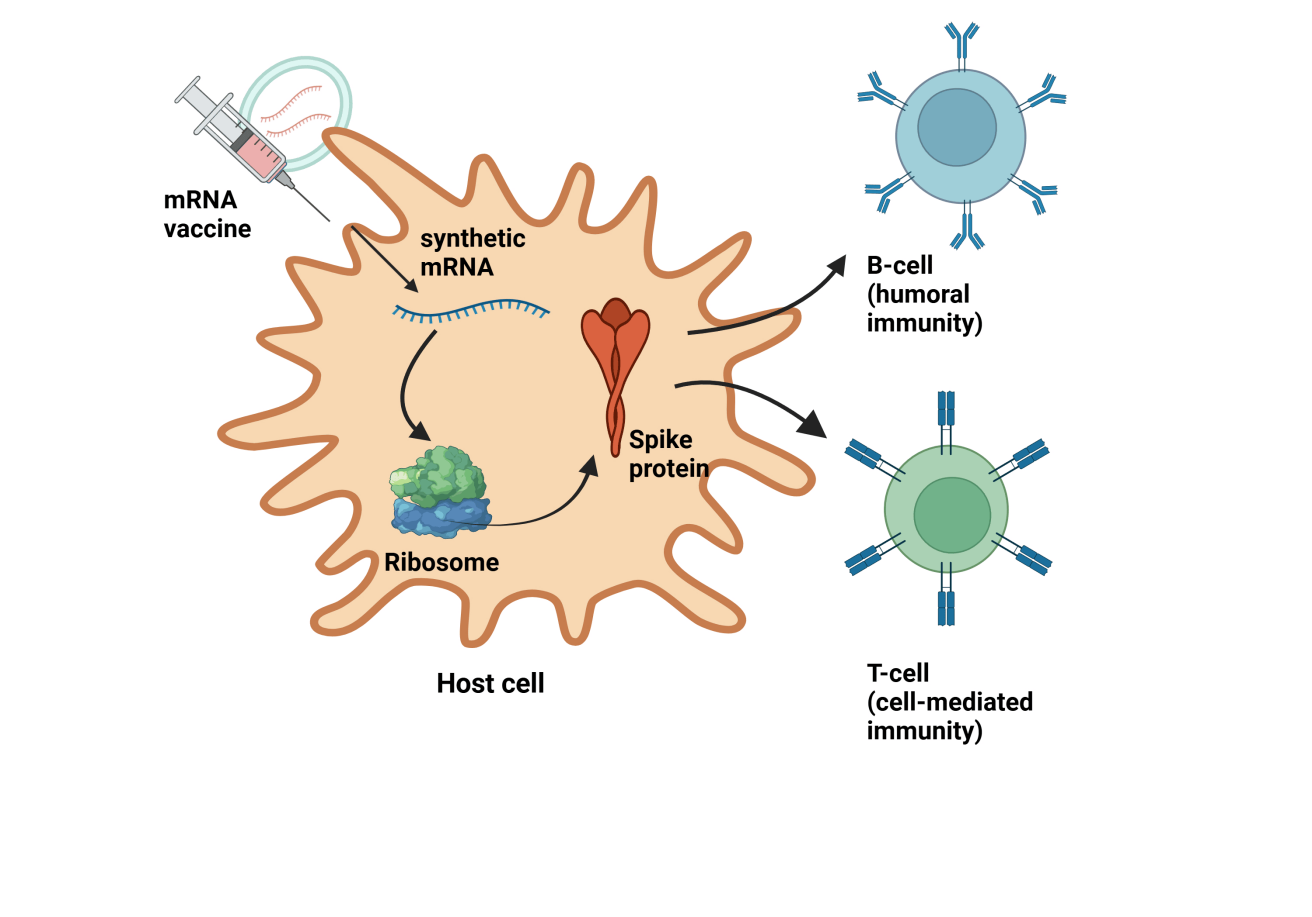
Mechanism of action of mRNA vaccines. Synthetic mRNA is delivered into the host cell via lipid nanoparticles. Ribosomes translate the mRNA into an antigen protein presented on the cell surface using MHC molecules. This presentation activates B-cells, which produce antibodies, and T-cells, which destroy infected cells. Created using Biorender.com MHC: Major histocompatibility complex, mRNA: Messenger ribonucleic acid

The operational mechanism of action of mRNA vaccines involves a series of cellular and immunological processes designed to generate a targeted immune response. It consists of the delivery of synthetic mRNA into host cells, typically facilitated by lipid nanoparticles (LNPs) that enhance cellular uptake and protect the mRNA from degradation [5,6]. Once inside the cytoplasm, the host's ribosomes translate the mRNA sequence into the corresponding antigenic protein, such as the spike protein of SARS-CoV-2 [7], which is then processed and presented on the cell surface via major histocompatibility complex (MHC) molecules. This endogenous production of antigen allows the immune system to recognize it as foreign, thereby initiating a comprehensive immune response. This response includes the activation of B-cells (humoral immunity), which produce specific antibodies, and T-cells (cell-mediated immunity), which target and destroy infected cells, establishing immunological memory that equips the body to combat future exposures to the actual pathogen [5-7]. Figure 1 illustrates the mRNA-mediated immune response.

## Review

Advantages of mRNA vaccines

mRNA vaccines offer several advantages over traditional vaccine platforms, making them a powerful tool for preventing infectious diseases. These benefits contribute to their rapid development, high immunogenicity, safety, scalability, and adaptability, positioning them as an essential solution for controlling both existing and emerging pathogens.

One significant advantage of mRNA vaccines is their high immunogenicity, which allows them to induce strong and long-lasting immune responses. mRNA vaccines encode pathogen-specific antigens, enabling the host’s cells to produce these antigens in their native conformation. This process effectively mimics natural infection, triggering both humoral and cellular immunity [2-7]. The ability of mRNA vaccines to activate T-helper cells, cytotoxic T cells, and B cells enhances the generation of neutralizing antibodies and memory responses, providing robust protection against infectious agents [2-7].

The safety profile of mRNA vaccines is another important advantage over traditional vaccine platforms. Conventional vaccines, such as live-attenuated and inactivated vaccines, carry risks associated with residual virulence or potential reversion to a pathogenic form [2-7]. 

Scalability and manufacturing efficiency are also key strengths of mRNA vaccines. Traditional vaccine production methods, such as those used for inactivated or protein subunit vaccines, often require large-scale cell culture systems, bioreactors, or chicken eggs, making production complex and time-consuming [2-8]. In contrast, mRNA vaccines can be synthesized in vitro using cell-free systems, allowing for rapid and large-scale production with fewer resource-intensive steps [9]. This feature has been particularly advantageous during pandemics, where the demand for vaccines far exceeds production capacity. The ability to quickly manufacture mRNA vaccines at scale ensures that more individuals can be vaccinated within a short period, reducing disease transmission rates and mitigating the public health burden of infectious outbreaks.

Another critical advantage of mRNA vaccines is their adaptability to pathogen variability, which is essential for addressing rapidly evolving viruses such as influenza and SARS-CoV-2. Traditional vaccines require constant reformulation and manufacturing updates to remain effective against mutating pathogens. In contrast, mRNA vaccine technology is modular, meaning that the vaccine sequence can be rapidly modified to match new viral variants [3-9]. This adaptability was evident during the COVID-19 pandemic, where updated mRNA vaccines targeting emerging variants (e.g., Omicron, Delta, and Beta) were developed within months of identifying new viral mutations [10].

The advantages of mRNA vaccine technology make it a versatile and powerful tool for addressing both current and future public health challenges [4]. The combination of rapid development, strong immunogenicity, high safety, scalability, and adaptability positions mRNA vaccines as a revolutionary breakthrough in vaccinology [2]. As researchers continue to optimize mRNA stability, delivery methods, and immune-enhancing adjuvants, the potential for universal vaccines that provide broad and long-lasting protection against infectious diseases becomes increasingly feasible. Future advancements in self-amplifying mRNA (saRNA), thermostable formulations, and next-generation nanoparticle delivery systems will further enhance the effectiveness and accessibility of mRNA vaccines, ensuring that they remain a cornerstone of global pandemic preparedness and disease prevention [11-12]. 

Universal vaccine design: strategies and innovations

The development of a universal vaccine aims to address the limitations of current vaccines, which often require frequent reformulation due to pathogen mutation and immune evasion. Unlike traditional vaccines that target highly variable regions of pathogens, universal vaccines focus on conserved antigens that remain stable across different strains, thereby offering broad-spectrum protection against a range of viral and bacterial infections [8]. mRNA technology provides a flexible and rapidly adaptable platform for such universal vaccines by enabling the precise encoding of highly conserved antigens. Several innovative strategies are currently being explored to develop mRNA-based universal vaccines, including targeting conserved antigens, utilizing self-amplifying mRNA (saRNA), and integrating artificial intelligence (AI) in antigen discovery and optimization [8,12].

Targeting Conserved Antigens

One of the biggest challenges in vaccine development is antigenic variability, particularly in viruses such as influenza, HIV, and coronaviruses, which rapidly mutate and develop immune escape mechanisms. Traditional vaccines typically target immunodominant epitopes, which are prone to frequent mutation, leading to vaccine escape and reduced efficacy. Universal vaccines circumvent this challenge by targeting conserved regions of viral proteins, which remain stable across different variants and strains [12].

In the case of influenza viruses, conventional vaccines primarily target the head domain of the hemagglutinin (HA) protein, which undergoes rapid mutation through antigenic drift and shift. However, research has shown that the HA stalk region is much more conserved and provides cross-protection against different influenza subtypes. Several mRNA vaccine candidates are now being designed to express HA stalk antigens, inducing broad-spectrum immunity without requiring frequent updates [3-12].

Targeting conserved internal viral proteins, such as nucleoproteins and polymerases, is another promising approach. These proteins tend to be highly preserved among viral families and elicit strong T-cell responses, which contribute to long-lasting immunity [13]. Researchers are also investigating cross-protective epitopes shared among multiple pathogens to develop broad-spectrum mRNA vaccines that can prevent entire families of viruses rather than just specific strains. 

Self-Amplifying mRNA (saRNA)

A major innovation in mRNA vaccine technology is the development of self-amplifying mRNA (saRNA), which significantly enhances antigen expression within host cells. Unlike conventional mRNA vaccines, which rely on external booster doses to maintain immunity, saRNA includes replicase enzymes, enabling the continuous production of antigenic proteins within the cell for an extended period. This process not only reduces the amount of mRNA needed per dose but also enhances immunogenicity, resulting in stronger and longer-lasting immune responses [13].

The ability of saRNA to generate high antigen levels with lower doses makes it a cost-effective and scalable solution for global immunization efforts. Traditional mRNA vaccines require relatively high doses to achieve protective immunity, which increases production costs and places strain on manufacturing capacities. In contrast, saRNA technology allows for dose-sparing strategies, meaning that a single batch of vaccines could protect more individuals, thereby improving vaccine accessibility in low-resource settings [12].

Beyond cost-effectiveness, saRNA vaccines show promise in inducing stronger and more durable immune responses. Preclinical studies have demonstrated that saRNA-based vaccines elicit robust memory B-cell and T-cell responses, which are essential for long-term protection [12]. Furthermore, because saRNA enables prolonged antigen presentation, it may reduce the need for booster doses, making vaccines more convenient for mass immunization programs.

Despite its advantages, saRNA technology faces challenges related to stability, delivery mechanisms, and potential immune responses against the replicase enzyme. Scientists are currently developing optimized lipid nanoparticle (LNP) formulations and chemical modifications to enhance the stability and delivery efficiency of saRNA vaccines [3-14]. Continued clinical research is needed to determine the ideal saRNA configurations that maximize immunogenicity while minimizing adverse effects.

AI and Machine Learning in Antigen Design

Artificial intelligence (AI) and machine learning algorithms are transforming the field of vaccine research by enhancing antigen discovery, predicting viral evolution, and optimizing vaccine formulations. AI-based models can analyze vast datasets of viral genomes, allowing researchers to identify conserved epitopes and predict future viral mutations, thereby enabling the design of broadly neutralizing vaccines [11-14].

One of the key applications of AI in mRNA vaccine development is the identification of broadly neutralizing antibody (bnAb) targets. These antibodies are capable of binding to multiple viral strains and preventing infection even in the presence of genetic mutations. AI-powered models can map out conserved regions of viral proteins, helping scientists select optimal antigen sequences for inclusion in universal mRNA vaccines [15]. This approach has already been applied in the development of universal influenza and coronavirus vaccines, significantly improving their efficacy against emerging variants.

Machine learning algorithms are also being used to predict immune responses and optimize mRNA vaccine design. By simulating host-pathogen interactions, AI can help researchers determine which antigenic regions are most likely to trigger strong immune responses. Additionally, AI can assist in designing stable mRNA sequences, improving delivery formulations, and minimizing unwanted immunogenic effects [12-15].

Another groundbreaking application of AI in vaccine research is real-time epidemiological surveillance. AI models can track genomic changes in circulating viruses, allowing scientists to preemptively modify mRNA vaccines before new variants become dominant [15]. This proactive approach has the potential to eliminate the need for annual vaccine updates, making universal vaccines a long-term reality rather than just an aspiration.

Despite these promising developments, the use of AI in mRNA vaccine design is still in its early stages, and further research is needed to refine predictive algorithms and optimize computational models. Challenges such as data biases, computational limitations, and validation of AI-generated antigens must be addressed before AI-driven vaccines can be widely implemented [2-15]. However, as AI technology continues to advance, it is expected to revolutionize the speed, accuracy, and effectiveness of universal vaccine development in the coming years.

Applications in combating the world’s deadliest diseases

Messenger RNA (mRNA) vaccine technology has demonstrated significant potential in addressing various high-risk pathogens, including influenza viruses, coronaviruses, and antimicrobial-resistant bacteria.​

Influenza

Influenza viruses are notorious for their high mutation rates, leading to frequent antigenic drift and shift, which necessitates annual updates of traditional vaccines. mRNA vaccines offer a promising alternative due to their rapid development and adaptability. Studies have shown that mRNA vaccines encoding hemagglutinin (HA) antigens can induce robust immune responses in animal models, providing protection against various influenza strains [2-15]. Additionally, self-amplifying mRNA (saRNA) platforms have been explored to enhance immunogenicity while reducing the required dosage, further improving the feasibility of universal influenza vaccination [13-15].​

Coronaviruses (SARS-CoV-2, MERS-CoV, SARS-CoV)

The COVID-19 pandemic underscores the need for broadly protective coronavirus vaccines. Research has identified cross-reactive T-cell epitopes that could serve as universal vaccine targets, reducing the severity of future outbreaks [12-15]. mRNA vaccines, such as those developed by Pfizer-BioNTech (New York, USA) and Moderna (Massachusetts, USA), have demonstrated high efficacy in preventing COVID-19 [16]. Beyond SARS-CoV-2, mRNA platforms are being investigated for their potential to target other coronaviruses, including Middle East respiratory syndrome coronavirus (MERS-CoV) and severe acute respiratory syndrome coronavirus (SARS-CoV), by encoding conserved viral antigens to elicit broad immunity [17]. This adaptability highlights the potential of mRNA vaccines in responding to current and future coronavirus outbreaks.

Antimicrobial-Resistant Bacteria

The rise of antimicrobial-resistant (AMR) bacteria poses a significant global health threat. mRNA vaccines have the potential to combat AMR pathogens by encoding bacterial antigens that elicit protective immune responses. For instance, recent studies have explored mRNA vaccines targeting *Clostridioides difficile*, a bacterium responsible for severe infections, with promising results in animal models [18]. This approach could be extended to other AMR bacteria, offering a new strategy to prevent infections that are difficult to treat with existing antibiotics.

Challenges and future directions

Despite the significant promise of mRNA-based universal vaccines, several key challenges must be addressed to ensure their widespread effectiveness and accessibility. While mRNA vaccines have demonstrated high efficacy against SARS-CoV-2, their application as universal vaccines for various infectious diseases is still evolving. The primary concerns include storage and stability, durability of immunity, and immune evasion by pathogens. To overcome these hurdles, future research should focus on thermostable formulations, adjuvant optimization, and AI-driven antigen discovery.

One of the biggest obstacles facing mRNA vaccines is their storage and stability. Currently, mRNA-based vaccines require ultra-cold storage conditions, with temperatures as low as -70°C for Pfizer-BioNTech's BNT162b2 (New York, USA) and -20°C for Moderna's mRNA-1273 (Massachusetts, USA) [9-18]. These stringent storage requirements make global distribution, particularly in resource-limited settings, extremely challenging. Many low-income countries lack the infrastructure to maintain ultra-cold chain logistics, resulting in delays or limitations in vaccine access [12-18]. Additionally, even in developed nations, logistical complications arise in maintaining consistent refrigeration from production to administration. Future research must prioritize the development of thermostable mRNA formulations that can be stored at refrigerated or ambient temperatures, ensuring greater accessibility in rural and underdeveloped regions [14-18]. Advances in lyophilization (freeze-drying) techniques and lipid nanoparticle stabilization are promising solutions for improving the temperature resilience of mRNA vaccines [2-18].

Another significant challenge relates to the durability of immunity provided by mRNA vaccines. While these vaccines have demonstrated strong initial immune responses, the longevity of protection remains uncertain [4-18]. Studies on COVID-19 mRNA vaccines indicate that antibody levels decline over time, prompting the need for booster doses to sustain protection against emerging variants [19]. This phenomenon raises concerns about whether universal mRNA vaccines will provide long-term immunity or require frequent booster shots, which could complicate global immunization efforts [16-19]. To address this issue, researchers are investigating optimized vaccine formulations that enhance memory B-cell and T-cell responses, thereby promoting long-lasting immune protection. Additionally, the inclusion of novel adjuvants, such as toll-like receptors (TLR) agonists and cytokine-based immune boosters, may enhance the duration of immune responses, reducing the need for frequent revaccination [20].

Immune evasion is another major concern that affects the efficacy of universal mRNA vaccines. Some pathogens, including influenza, HIV, and coronaviruses, possess high mutation rates, allowing them to escape immune recognition [11-20], requiring continued antigen optimization [20]. For example, HIV has developed extensive glycan shielding mechanisms that prevent immune cells from effectively neutralizing viral particles, posing a major challenge for vaccine development [10-20]. Similarly, influenza viruses undergo antigenic drift and shift, necessitating frequent updates to existing vaccines to maintain efficacy [12-20]. Universal vaccine strategies must therefore focus on identifying highly conserved viral epitopes, ensuring that mRNA vaccine-encoded antigens remain effective against diverse viral strains. The integration of computational modeling, artificial intelligence (AI), and structural biology can facilitate the discovery of broadly neutralizing antibody (bnAb) targets, which may significantly improve vaccine durability and efficacy [11-22].

Future research directions should prioritize developing thermostable mRNA vaccines, optimizing adjuvants to enhance long-term immunity, and expanding AI-driven antigen discovery. Thermostable formulations will increase vaccine accessibility worldwide, especially in low-resource environments, while adjuvant optimization will ensure that vaccines stimulate robust and sustained immune responses. By leveraging artificial intelligence (AI) and bioinformatics, researchers can identify conserved epitopes across multiple virus strains, leading to the design of broad-spectrum mRNA vaccines capable of protecting against multiple variants and even entire virus families [15]. Moreover, AI-driven approaches can significantly accelerate the identification of conserved antigenic targets, allowing for the development of next-generation universal vaccines that remain effective against rapidly evolving pathogens [15-22]. Addressing these challenges will be crucial in realizing the full potential of mRNA-based universal vaccines, paving the way for broad-spectrum protection against future pandemics.

Limitations of the review

While this review highlights the potential of mRNA-based universal vaccines, it is important to recognize certain limitations. The discussion relies heavily on conceptual frameworks and emerging technologies that, in many cases, have not yet undergone extensive clinical validation. Many of the approaches outlined, such as self-amplifying mRNA, AI-driven antigen selection, and nanoparticle delivery systems, are still in the experimental or early implementation stages. As a result, some assessments are based on projected capabilities rather than proven outcomes. In addition, the focus of this review is primarily on viral pathogens, with less emphasis on non-viral targets such as parasites or broader applications beyond infectious disease. The rapid pace of research in this field also poses a challenge, as new developments may arise faster than they can be comprehensively analyzed. Lastly, the review is limited by the availability of open data on proprietary platforms and vaccine formulations, which restricts a full evaluation of current and future candidates.

## Conclusions

Messenger RNA (mRNA) vaccine technology marks a significant shift in immunology, enabling rapid and adaptable responses to emerging infectious threats. Its modular design and streamlined production allow for the swift development of vaccines tailored to novel pathogens, making it a vital tool for global health preparedness. The promise of mRNA-based universal vaccines lies in their ability to generate broad and durable immune responses by targeting conserved antigens across diverse viral and bacterial families. Advancements such as self-amplifying mRNA, innovative delivery systems, and computational antigen prediction enhance both the precision and scalability of this platform. These developments accelerate vaccine production and improve the potential for cross-protection against multiple variants and pathogen subtypes.

Nonetheless, several challenges must be addressed to fully realize the potential of mRNA-based universal vaccines. These include issues of molecular stability, cold chain dependency, and inconsistent long-term immune responses. Continued research and innovation are essential to develop thermostable formulations and enhance immune-stimulating components, thereby improving global accessibility, particularly in resource-limited regions. As the field advances, the integration of synthetic biology, nanotechnology, and artificial intelligence is opening new pathways for next-generation vaccine design. With ongoing refinement, mRNA platforms are well-positioned to become a cornerstone of proactive global health strategies, contributing to more resilient and equitable public health systems.
